# Protein A-like Peptide Design Based on Diffusion and ESM2 Models

**DOI:** 10.3390/molecules29204965

**Published:** 2024-10-21

**Authors:** Long Zhao, Qiang He, Huijia Song, Tianqian Zhou, An Luo, Zhenguo Wen, Teng Wang, Xiaozhu Lin

**Affiliations:** 1 Department of Pharmaceutics, Beijing Institute of Petrochemical Technology, Beijing 102627, China; tom@bipt.edu.cn (L.Z.); heqiang0889@163.com (Q.H.); luoan@bipt.edu.cn (A.L.); wenzhenguo@bipt.edu.cn (Z.W.); 2 Department of Computer Science, Beijing Institute of Petrochemical Technology, Beijing 102627, China; songhuijia0224@163.com (H.S.); zhoutq0619@163.com (T.Z.)

**Keywords:** protein design, diffusion model, ESM2, peptide screening, biological activity

## Abstract

Proteins are the foundation of life, and designing functional proteins remains a key challenge in biotechnology. Before the development of AlphaFold2, the focus of design was primarily on structure-centric approaches such as using the well-known open-source software Rosetta3. Following the development of AlphaFold2, deep-learning techniques for protein design gained prominence. This study proposes a new method to generate functional proteins using the diffusion model and ESM2 protein language model. Diffusion models, which are widely used in image and natural language generation, are used here for protein design, facilitating the controlled generation of new sequences. The ESM2 model, trained on the basis of large-scale protein sequence data, provides a deep understanding of the context of the sequence, thus improving the model’s ability to generate biologically relevant proteins. In this study, we used the Protein A-like peptide as a model study object, combined the diffusion model and the ESM2 model to generate new peptide sequences from minimal input data, and verified their biological activities through experiments such as the BLI affinity test. In conclusion, we developed a new method for protein design that provides a novel strategy to meet the challenges of generic protein generation.

## 1. Introduction

Proteins are fundamental molecular building blocks essential to all living organisms. These biomolecules consist of linear sequences of amino acids that fulfill their functions through complex three-dimensional structures. It is because the structure of a protein determines its function that scientists have invented nuclear magnetic resonance (NMR) techniques, X-ray techniques, and Cryo-ET to determine the structure of proteins [1,2,3,4]. This laid the foundation for subsequent protein design. In order to make proteins suitable for specific scientific applications, engineers have employed a range of experimental and computational strategies aimed at creating different amino acid sequences to achieve specific structures and functions [5].

The resolution of protein structure lays a solid foundation for subsequent protein design. However, experimental techniques alone can no longer meet the demand for rapid design and engineering. Therefore, scientists have gradually turned to computational methods in combination with experimental data to explore more efficient protein design strategies. Computational protein design mainly follows two parallel development paths: design methods based on evolutionary principles and design methods based on artificial intelligence. The former is motivated by increasingly faster sequencing and homology detection methods, which have been developed by creating large sequence databases (e.g., UniProt [6] and the Protein Data Bank, or PDB [7]) and further building the databases into evolutionarily relevant protein families (e.g., PFAM [8]). With these databases, the next step is to retrieve homologous sequences from the sequence databases, starting from amino acid sequences, in order to retrieve homologous sequences and construct multiple sequence alignment (MSA) [9]. MSA has a variety of evolutionary models including conservatism reflecting structural and functional constraints, pairwise co-evolution, and higher-order co-evolution [10]. There are also early models such as position-specific scoring matrices (PSSMs) that focus only on capturing the amino acid frequencies of a specific site. Rosetta3 is a traditional protein design software based on physical and energetic functions capable of designing new proteins by calculating the protein structure and energy optimization [11]. The main limitations of this protein design method are high computational cost, wide search space, energy function limitations, difficulty in designing proteins with new functions, and insufficient generalization ability to effectively address the design needs of complex proteins.

In response to the limitations of experimental techniques, computational and artificial intelligence methods are rapidly emerging in protein design. These models are capable of predicting proteins’ properties from their sequence, structure, or both to further guide protein design [12]. Examples include the emergence of Alphafold2, which is capable of predicting a protein’s structure from its sequence [13], and Alphafold3, which not only predicts protein structure but also performs protein docking tasks [14]. However, despite the remarkable progress these technologies have made in protein structure prediction and interaction understanding, designing or generating proteins on demand still faces complex challenges. In recent years, a series of important works in David Baker’s lab have successfully realized generative protein design [5]. For example, proteinMPNN surpassed Rosetta in terms of sequence recovery rate, multi-strand coupled design, and success rate of experimental validation, demonstrating its excellence in a wider range of protein design applications [15]. One of the most significant contributions is the construction of protein generation models through deep learning.

Early generative models such as variational autoencoders (VAEs) efficiently construct and infer complex data distributions by learning the latent properties of the input data [16], which are widely used in the field of unsupervised learning, including protein sequence design. Hawkins Hooker utilized variational autoencoders (VAEs) trained on nearly 70,000 luciferase-like oxidoreductases to generate functional luxA bacterial luciferase variants, successfully demonstrating their utility [17]. Both aligned (MSA-VAE) and original sequence (AR-VAE) models reproduced family-specific amino acid patterns, with MSA-VAE capturing long-range dependencies associated with 3D structures. Experimentally validated variants showed that 21 out of 23 variants of MSA-VAE and 18 out of 24 variants of AR-VAE retained measurable luminescence, confirming the validity of the models in protein design.

VAE penalizes the model by learning the probability distribution of the data and minimizing the KL dispersion so that the generated data points are close to the distribution of real data points. And another generative model, the Generative Adversarial Network (GAN) [18], learns the probability distribution of the data through an adversarial game between the generator and the discriminator. ProteinGan is morphed from GAN and also consists of a generator and a discriminator [19]. ProteinGan learns how a protein can evolve from a multidimensional amino acid sequence space and creates new sequence variants with natural physical properties. Using malate dehydrogenase as a model, experiments show that 24% of the protein is soluble. This shows that ProteinGan can rapidly generate completely new proteins with functionality. As you can see, scientists have begun to use generative models in the protein field to generate the proteins they want. Although the positional entropy of the set of sequences generated by ProteinGan matches the positional entropy of the initial input fairly well, these sequences have been extended to new structural domains in CATH classification [20], showing the structural diversity of the results.

Although MSA-VAE and ProteinGan show great potential in protein design, they still face challenges in handling complex protein structure generation, especially in protein generation tasks that require the precise control of protein 3D structures. Therefore, researchers have developed the diffusion-based RfDiffusion model (denoising diffusion probabilistic models, DDPM) [21,22]. RfDiffusion combines structure prediction (RoseTTAFold) with diffusion model generation [23] to design proteins from scratch. This approach allows the creation of protein structures with complex functional properties. The model excels at a variety of protein design challenges, including the design of monomers, binders, and symmetric oligomers. It also allows for specific scaffolds and unconditional protein generation, meeting a wide range of research and practical applications in biomedicine and chemistry.

To design proteins more efficiently, it is not enough to generate sequences. We also need to gain insight into how sequence determines protein structure and function, and protein language models provide insight into sequence–structure–function associations by learning from a large amount of known protein data. An example is ESM2, a protein language model based on the Transformer structure [24,25,26]. ESM2 utilizes unsupervised learning to train deep-language models directly on a huge dataset containing 250 million natural protein sequences, including 8.6 billion amino acids. This approach allows the model to capture rich, biologically relevant features without the need for labeling data. The model learns a multi-scale representation of the protein sequence, capturing details of a wide range of patterns from biochemical properties of amino acids to distal homology. This comprehensive learning contributes to a deeper understanding of protein functions and relationships. ESM2 addresses this problem by learning contextual representations, analogous to how natural language processing models infer word meanings from context. This analogy has proven useful in understanding complex protein biodata [27,28].

Although the previously mentioned models have successfully enabled sequence-based and structure-based protein design and generation, they typically rely on large datasets and often utilize structure-based information such as multiple sequence alignment (MSA) or RoseTTAFold for protein generation. In this paper, we propose an innovative approach to optimizing the functional properties of proteins by using fewer protein sequences for protein generation through diffusion modeling and then parameterizing the generated proteins in combination with ESM2 modeling. Next, we screen these proteins and validate their biological activities by laboratory wet experiments. To ensure the validity of our approach, a specific target Protein A was selected as the prototype of the generated proteins in this study. With this strategy, we aim to reduce the dependence on large-scale datasets while improving the accuracy and utility of the generated proteins, bringing new research ideas and application possibilities to the field of protein engineering.

## 2. Results

We converted the protein into a picture, and by inputting the picture into the model for the diffusion process, each time step corresponds to a result of the diffusion process. Then, we can obtain a new picture, convert the new picture into a new protein sequence, and successfully obtain a new sequence. Since we used the Protein A protein design as the basis, the protein picture we input is Protein A, so the generated protein sequences will all have the same sequence length as Protein A. As for the number of Protein A-like protein sequences generated each time, it completely depends on the length of the time step we set in the algorithm, because a new result will be output for each time step.

For a series of protein sequences generated by the diffusion model mimicking Protein A, we used the AlphaFold2 technique to obtain the tertiary structures of these new protein sequences and screened a batch of protein sequences based on the characteristic distances, computed from the protein-embedding vectors obtained using the ESM2 model (The greater the characteristic distance, the smaller the similarity between the two proteins is likely to be.); the skeleton distances, predicting the tertiary structure of the proteins using Alphafold2 and then using the PyMol for calculation (The smaller the distance between the backbones, the greater the structural similarity between the two proteins.); and solubility, using the online program made public by NovoPro, an online website for protein solubility prediction (Solubility is a predicted value that ranges from 0 to 1. A value closer to 1 means a higher predicted solubility. Therefore, we used the solubility of Protein A as our benchmark here.). A batch of protein sequences was screened as shown in the Table 1.

In addition to this, we used state-of-the-art protein generation models for sequence generation in the Protein A-like generation stage. These include the ESMIF [29] model based on the Facebook institute, which is a protein design model based on the ESM protein language model; the proteinMPNN model from David Baker’s lab, which is a backbone-based protein design model; and the RfDiffusion model from David Baker’s lab, which is a structure-based denoising model. Z5 is the sequence generated with the ESMIF model, Z6 is the sequence generated with proteinMPNN, and Z7 is the sequence generated with RfDiffusion.

**Figure 1:**
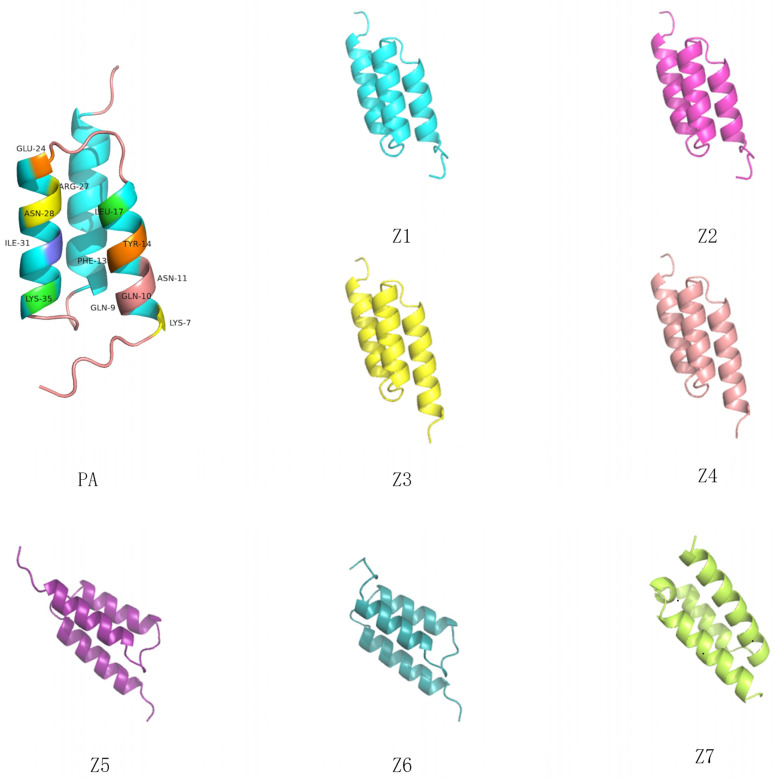
Tertiary structure of Protein A (PA) and the newly generated protein sequences Z1–Z4. Z5, Z6, and Z7 are from the ESMIF, proteinMPNN, and RfDiffusion models, respectively. Using AlphaFold2 to predict the tertiary structure of proteins, we display the tertiary structure of the protein using PyMOL for the resulting PDB files and perform alignment operations.

In terms of feature distance, skeleton distance, and solubility compared, the protein sequences generated by the other three models (Z5–Z7) and the sequences generated by our current experiment (Z1–Z4) are all very different. The solubilities of Z5–Z7 are all much lower than the solubility of the original Protein A; from the feature distances and backbone distances, we can also see that they are very different from the original Protein A; and the backbone distances of Z7 are very different from Protein A. There is a difference of 7 Å units, in fact. From the protein structure diagram in Figure 1, we can also see that the structure of Z7 is far from Protein A (PA), so Z5–Z7 would not be our candidates, and we decided to send our generated protein sequences (Z1–Z4) to the lab for the next experiments.

The experimental data showed that the binding and dissociation of the Protein A parental sequence, Protein A-Z1, and mAb1 were almost the same, and the affinity of Protein A-Z1 was almost the same as that of the Protein A parental sequence, reaching E-10, which is a great help for the subsequent study. Table 2 displays the test results, and Supplementary Material Figure S1 plots the kinetic curves for Protein A with Z1–Z4.

As can be seen from the data in Table 2 and the kinetic curves in Supplementary Material Figure S1, the Kd values of Protein A-Z1, Protein A-Z2, and Protein A-Z4 are in the same order of magnitude as those of Protein A-parental, and all of them reach the impressive picomolar (pM) level. Among them, Protein A-Z1 was particularly impressive, with a Kd value of 2.58 × 10^−10^ M, which is almost comparable to Protein A-parental. In contrast, the affinity performance of Protein A-Z3 was inferior, with a Kd value of 1.77 × 10^−9^ M, which is an order of magnitude different from Protein A-parental and shows a lower binding capacity. Based on the comparison of these Kd values, Protein A-Z1 is undoubtedly the closest candidate to Protein A-parental, exhibiting extremely high affinity and thus being more suitable for subsequent in-depth research and application development, whereas Protein A-Z3 may require further optimization.

## 3. Discussion

Johnson et al. have done a lot of basic and pioneering research work in this field; in the literature [30], they performed three rounds of model optimization, completed 500+ protein expression and purification experiments of natural and generated sequences, and finally obtained a filtering standard, which increased the experimental success rate by at least 50%. As for our research, one of the important ideas is to use natural proteins as the target sequences, which mainly stems from our latest understanding of generative modeling: the essence of generative modeling is mimicry, the highest level of mimicry is the limit approximation, and the natural proteins as the target sequences are our limit. Based on this idea, we found synthetic proteins Z1 and Z2 with similar functions to parental Protein A among the four selected generative sequences, and their effectiveness can be compared with Johnson et al.’s experiments.

The protein sequences (Z5–Z7) generated by the other models show the differences between the sequences generated by our calculations and those generated by this experiment. These models (ESMIF, proteinMPNN, RfDiffusion) have actually achieved good performance in the field of general protein design, but they may not be able to achieve a more controllable step-by-step generation for a specific protein sequence simulation. The RfDiffusion model is also a protein generation model based on protein structure diffusion, which is different from our diffusion model in this design in that we use a sequence-based diffusion model, where we convert the sequence into a grayscale map first, and then carry out the diffusion process for denoising and generating the protein sequence. And because we use sequence-based design, each time we obtain a new sequence, although the difference between the original sequence and the new sequence is relatively small, we can still verify its biological activity through the wet test. However, we cannot be as good as the other model, which instantly generates the biological activity of the protein sequence, and then goes to the verification of its biological activity. But our model can guarantee that each time the result will be more or better bioactivity than the previous sequence (this statement is similar to the gradient descent in deep learning), and then find the optimal solution. Finding that optimal protein sequence, we use a similar idea.

Despite the remarkable results achieved in this experiment, we also acknowledge the limitations of the existing models and methods, which need to be further optimized and improved. The functions of proteins are closely related to their structures, and their functions can be inferred from their affinities; therefore, in our subsequent studies, we will continue to explore how to analyze and design protein sequences more deeply from both structural and functional dimensions. It is hoped that we can find synthetic sequences with affinities beyond PA, which will help us to accurately design and predict proteins with specific functions and structures, and thus, play a greater role in the biomedical field.

## 4. Materials and Methods

### 4.1. Diffusion Denoising Probabilistic Model (DDPM)

The DDPM model mainly consists of a forward process, which continuously adds noise to the original image, and a backward process, which recovers the image by continuously denoising it.

**Figure 2:**
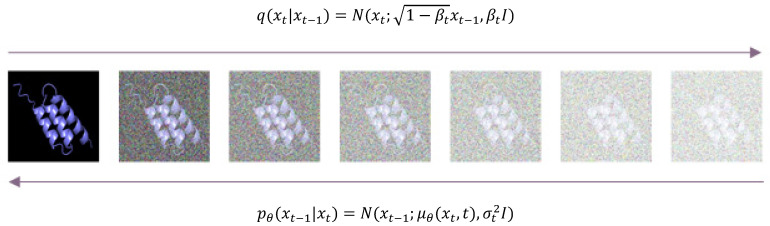
DDPM model. The image illustrates the forward and reverse processes in a diffusion model. The forward process adds Gaussian noise $\beta_{t}$ to the data, the formula describes the diffusion process from $X_{t-1}$ to reach $X_{t}$, where $X_{t}$ is a Gaussian distribution given $X_{t-1}$. Its mean is $X_{t-1}$ multiplied by the coefficient $\sqrt{1-\beta_{t}}$, and its variance is $\beta_{t}\;I$, where *I* is the identity matrix. The reverse process aims to denoise and reconstruct the original data. The formula at the bottom describes the generation process, that is, from $X_{t}$ to $X_{t-1}$. $X_{t-1}$ follows a Gaussian distribution with a mean of $\mu_{\theta }\left(X_{t},t\right)$ calculated by a neural network (parameter θ) and a variance of $\sigma_{t}^{2}\;$*I*.

It should be declared here that Figure 2 illustrates only the process of adding and denoising the diffusion model from the point of view of image visualization, and the real protein sequence diffusion model is built on the one-hot image:

The diffusion process takes DDPM in the previous step $x_{t-1}$ and applies random noise to get the result of the next step $x_{t}$, that is, $x_{t}=\sqrt{\alpha_{t}}x_{t-1}+\sqrt{1-\alpha_{t}}\varepsilon_{t}$ (For the detailed formula, see Supplementary Material Table S1). This process is equivalent to the process of obtaining a randomized noise from an ensemble with a mean of $\sqrt{\alpha_{t}}x_{t-1}$, and the variance is $\left(1-\alpha_{t}\right)I$ of the Gaussian distribution sampled. Eventually, the formula for adding noise can be obtained:

$$q(x_{t}|x_{0})=N\left(x_{t};\;\sqrt{{\bar{\alpha }}_{t}}x_{0},\;\left(1-{\bar{\alpha }}_{t}\right)I\right)$$

U-Net is used as the model structure, and the Kullback–Leibler (KL) scatter loss function is used to train the model. The training algorithm is as follows:

In the inverse process, given noisy samples, the image is recovered by predicting the noise level and denoising. (For the detailed formula, see Supplementary Material Table S2.) Assuming that the conditional probability distribution for the inverse is also Gaussian and that the Gaussian distribution actually has only two parameters, mean and variance, the neural network needs to compute, in effect,

$$q\left(x_{t-1}|x_{t}\right)∼N\left(x_{t-1};\mu_{t}\left(x_{t}\right),\sigma_{t}^{2}\right)=N\left(x_{t-1};\frac{1}{\sqrt{\alpha_{t}}}\left(x_{t}-\frac{1-\alpha_{t}}{\sqrt{1-\bar{\alpha }t}}\epsilon \right),\frac{\left(1-\bar{\alpha_{t-1}}\right)\left(1-\alpha_{t}\right)}{1-\bar{a_{t}}}I\right)$$

This inverse process cannot be derived directly, so we use a neural network to fit this distribution. Using an understanding of the inverse process, a model trained with forward diffusion is used to predict the noise added to the image. Having real and predicted noise turns this into a supervised machine learning problem.

**Figure 3:**
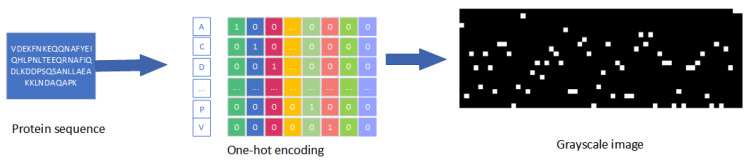
The process of encoding a protein into an image. Protein sequences are transformed into grayscale images by representing amino acids with one-hot encoded binary vectors, enabling their use in image-based analysis and machine learning models.

In the classical diffusion model, the object of processing is usually an image, and in this experiment, we applied this process to the generation of proteins. When dealing with protein sequences, it is first necessary to encode the protein sequences uniquely and thermally in order to be able to represent the protein sequences in a binary image, as shown in Figure 3.

The diffusion model’s forward process is to train a neural network that is intended to be used to predict noise. To obtain such a noise-prediction network, we intentionally use noisy images as inputs and noise as outputs and limit the noise to Gaussian noise, which ensures perfect mathematical derivation.

In the inverse process of the diffusion model, after adding noise, we use the gray map of the protein sequence and the time step *t* as inputs to the U-Net model for noise prediction. This predicted noise is then used in the denoising process. However, the nonlinearity and complexity of the neural network prevent the model from completely removing the noise. In the final stage of denoising, the model often retains a portion of the noise in the input. This part of the noise can be regarded as the “modification” of the original protein sequence to generate a new protein sequence.

### 4.2. ESM2 Model

Protein language modeling evolutionary scale modeling, or ESM2, is a model that was created by the FAIR Institute (Facebook AI Research) to predict the structure and function of proteins. It is pre-trained for biological sequences like amino acid sequences of peptides or proteins. The model is based on the Transformer architecture and is unsupervised and pre-trained on a large-scale database of protein sequences. By learning the evolution law of protein sequences and the sequence–structure–function relationship, the ESM2 model can generate its corresponding hidden vectors according to the protein sequences to represent its structure and function; it can also utilize the hidden vectors to accomplish a variety of bioinformatics downstream tasks, such as protein structure prediction, protein function annotation, protein sequence alignment, interaction analysis, etc. The ESM2 truly achieves the goal of using a deep learning model to learn protein sequences like a natural language, which is a powerful and universal protein language model that provides unique vision and new tools for protein science research. In order to better understand the generation process of ESM2, we retrace the history of generators from the early variational self-coding VAE [16], to the adversarial generative network GAN [18] in 2014, to the diffusion model DDPM [21] in 2020.

**Figure 4:**
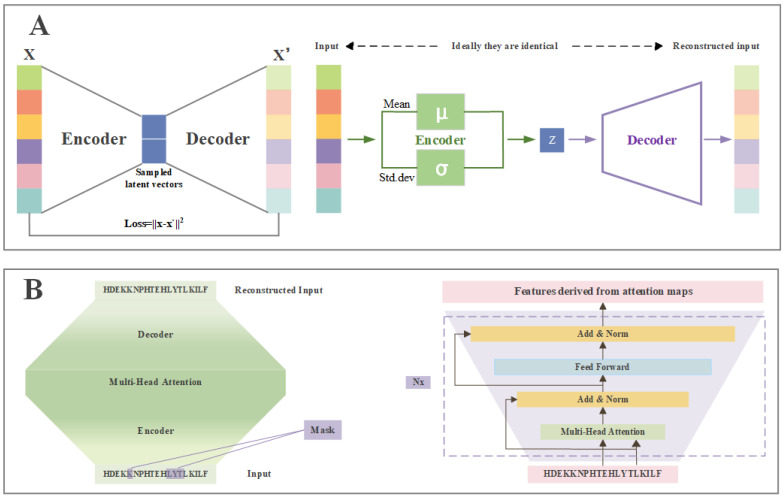
Methodology model structure. (**A**) is the VAE structure, and (**B**) is the Transformer and ESM2 structure. (**A**) This panel demonstrates a variational autoencoder structure where the input sequence is encoded into a latent space with mean (μ) and standard deviation (σ) parameters, which are then sampled to generate new sequences via the decoder. (**B**) The bottom left panel showcases a sequence-to-sequence model with attention, where an encoder processes a protein sequence, and a decoder reconstructs the input sequence, leveraging multi-head attention mechanisms to enhance feature extraction.

As shown in Figure 4A, it is a VAE model with a long drum structure in North Korea. The waist is thinner because the encoder at the bottom compresses and encodes the input sequences, and the decoder at the top completes the decoding recovery function so that the output is equal to the input, i.e., reconstruction. The right side of the hourglass shows a detailed unfolding structure diagram of the structure, which, after being trained and stabilized on large-scale databases, is generally used with the decoder part as the generator, the input noise type hidden vector Z, and then through the decoder network to obtain the generated sequence with specific semantics.

As shown in Figure 4B, for a diamond-shaped structure of the Transformer pre-training model, the waist is thickened because the encoder at the bottom upscales the input considering the multi-head attention mechanism, and then reconstructs it by downscaling the decoder at the top. Please keep in mind that the Transformer model is essentially an upgraded version of the VAE model, which includes only details such as positional embedding and self-attention mechanisms compared with the VAE model. The input part can be with or without a mask, and the output is a sequence without a mask, so that the pre-trained Transformer model has a prediction function. If we take the encoder alone, we can obtain the ESM2 model. When the input sequence passes through the encoder network, we can obtain the important features of that sequence. Especially after the pre-training of the 250 million protein databases, the ESM2 model obtained already contains the structural and functional features of the protein after evolution. For detailed parameters of the ESM2 model, see Supplementary Material Table S3. Supplementary Material Table S3 lists the number of layers in the ESM2 model and the corresponding parameter sizes and embedding dimensions. For example, when the protein sequence length is 65 amino acids, the embedding dimension of ESM2-650M is 1280, and the embedding dimension of ESM2-3B is 2560. The ESM2 model used in this paper is ESM2_t12_35M_UR50D, with the number of layers being 12, the parameter size being 35 M, and the embedding dimension being 480. This embedding representation captures the important protein sequence properties and also reveals the contextual relationships of the protein sequence.

In order to assess the characteristic properties of the newly obtained proteins, we input the generated sequences into the ESM2 model and calculate the characteristic distances between the two relatives to the target sequence. Of course, it is also possible to use the AlphaFold2 technique to obtain the backbone distances between the new sequence and the target sequence, thus ensuring their similarity in terms of physicochemical properties and structure, which is beneficial for our stemming screening of the synthesized sequence.

### 4.3. Purification and Expression of Proteins

Affinity assay of Protein A parents and predicted derivatives.

The parental sequence of Protein A and the Protein A-like derivatives obtained from the virtual prediction screening were repeated in tandem four times. The His tag was added at the C-terminal end, cloned into pET28a, and the expression plasmid was transformed into BL21(DE3) expression bacteria, which were cultured at 220 rpm on a shaking table, and the OD of the bacterial liquid was reduced to 0.6 by the addition of IPTG (the final concentration of which was 2 mmol/L) at 37 °C. After centrifugation and ultrasonication, the bacteria were collected and crushed, and the samples were sent to a Ni-NTA column, eluted by PBS + 500 mM imidazole. The purified proteins were dialyzed into 1 × PBS, and the purity of SDS-PAGE was more than 95%, so we performed subsequent affinity experiments using purified protein samples.

### 4.4. Affinity Determination

The affinity between purified Protein A and its derivatives Z1–Z4 and the human IgG4 monoclonal antibody mAb1 was tested using ForteBio Octet K2. It was specifically purified Protein A and its derivatives, Z1–Z4, that were fixed with a His sensor (Item No. 18-0038) at a concentration of 4 μg/mL for 120 s. After letting the sensor settle in PBST for 120 s, it was put into mAb1 solutions with concentrations of 50 nM, 25 nM, 12.5 nM, and 6.25 nM. The binding lasted for 120 s, and then the sensor was put back into PBST for 180 s to break the bond. The sensor was regenerated in glycine solution at pH = 1.7 for the next round of measurement.

## 5. Conclusions

In this paper, we first used the diffusion network model to simulate the generation of protein sequences and then encoded these sequences into hidden vectors using the ESM2 protein language model to calculate the distances between the protein backbones. Moreover, we also carried out solubility prediction and tertiary structure prediction of the screened proteins, which were used as an important basis for the screening. These steps not only improved our screening efficiency but also verified the effective binding of the screened proteins to the human IgG4 monoclonal antibody mAb1 by the ForteBio Octet K2 affinity test. The experimental data showed that these proteins showed striking similarities to the Protein A parental sequences in terms of key performance indicators such as affinity, binding rate, and dissociation speed. The traditional screening process requires people to randomly mutate sequences, and for all mutated sequences, affinity validation needs to be performed from scratch. However, this work is considered a waste of a lot of manpower, money, and time costs in the laboratory. If we use our generation and screening methods, it will greatly increase the efficiency and save manpower, money, and time costs in the laboratory. Because our experiments do not require a large number of context-specific protein datasets and are based on the diffusion model, rapid experiments can be realized, and more desirable protein sequences can be obtained in a short period of time. This highlights the potential of these proteins in biomedical applications. The success of this experiment not only confirms the effectiveness of the design and screening strategy adopted but also provides a solid technical foundation for future protein engineering and drug development.

## Figures and Tables

**Table 1:** Mimicking PA computer-generated protein sequences.

Number	Sequence	Characteristic Distance	Skeleton Distance (Å)	Solubility
PA	ADNKFNKEQQNAFYEILHLPNLNEEQRNGFIQSLKDDPSQSANLLAEAKKLNDAQAPK	-	-	0.814
Z1	VDNKFNKEQQNAFYEILHLPNLTEEQRNAFIQALKDDPSQSANLLAEAKKLNDAQAPK	0.02584	0.131	0.814
Z2	VDNKFNKEQQNAFYEILHLPNLTEEQRNAFIQDLKDDPSQSANLLAEAKKLNDAQAPK	0.02262	0.138	0.864
Z3	VDAKFDKEAQEAFYEILHLPDLTEEQRNAFIQDLKDDPSVSKAILAEAKKLNDAQAPK	0.02832	0.198	0.878
Z4	VDAKFDKEAQEAFYEILHLPDLTEEQRNAFIQNLKDDPSVSKAILAEAKKLNDAQAPK	0.02733	0.197	0.878
Z5	GPLGSSAEAQQARQEIQNLPNLQSQQLRQQFLQQLQQQPQQAQQLLQQAQQLNQQLQPP	0.03998	0.571	0.548
Z6	APDAFDPAARAAEAEIRALPHLRDPALRDAFLAALRADPAAAAALLAEARALNAALAPR	0.05185	0.596	0.565
Z7	PDPAALAELQNAFYEILHLPSATSPALRAAVLAALALPIDEALAFFRALRAALAAAAAA	0.04992	7.482	0.565

**Table 2:** $K_{D}$ values of Protein A and its mutants.

Number	$K_{D}\;\left(M\right)$	Kon (1/Ms)	Koff (1/s)	$Full\;\;R^{2}$
Protein A-parental	2.57 ± 0.15 × 10^−10^	5.49 × 10^5^	1.41 × 10^−4^	0.9981
Protein A-Z1	2.58 ± 0.11 × 10^−10^	1.15 × 10^6^	2.95 × 10^−4^	0.9916
Protein A-Z2	3.35 ± 0.14 × 10^−10^	1.15 × 10^6^	3.84 × 10^−4^	0.9873
Protein A-Z3	1.77 ± 0.30 × 10^−9^	5.49 × 10^5^	9.71 × 10^−4^	0.9936
Protein A-Z4	7.31 ± 0.23 × 10^−10^	5.20 × 10^5^	3.80 × 10^−4^	0.9966

## Data Availability

Code is available at https://github.com/tomlongcool/diffusion4Protein (accessed on 1 May 2024).

## Supplementary Materials

The following supporting information can be downloaded at: https://www.mdpi.com/article/10.3390/molecules29204965/s1, Figure S1: Kinetic Association-Dissociation Curve.; Table S1: Forward diffusion process. Table S2: Reverse diffusion process. Table S3: Parameters of the ESM2 model.

## Abbreviations

The following abbreviations are used in this manuscript:

MSA	Multiple Sequence Alignment
PSSM	Position-Specific Scoring Matrices
ESM	Evolutionary Scale Modeling
DDPM	Denoising Diffusion Probabilistic Models
GAN	Generative Adversarial Network
VAE	Variational Autoencoder
