# Supplementary material for: Protein A-like Peptide Design Based on Diffusion and ESM2 Models
Source: Molecules. 2024 Oct 21;29(20):4965. doi: 10.3390/molecules29204965 (PMC11510650; doi:10.3390/molecules29204965)
Supplement: Supplementary file 1 [file molecules-29-04965-s001.zip › molecules-3221540-supplementary.pdf]

Table S1. Forward diffusion process.

**Algorithm 1 Training**
Repeat
$x_0 \sim q(x_0)$
$t \sim Uniform(\{1, \dots, T\})$
$\epsilon \sim N(0, I)$
Take gradient descent step on $\nabla_\theta \| \epsilon - \epsilon_\theta(\sqrt{\bar{\alpha}_t} x_0 + \sqrt{1 - \bar{\alpha}_t} \epsilon, t) \|^2$
until converged

Firstly, it is the initial data sample x_0, is drawn from the data distribution $q(x_0)$. This is the starting point for the diffusion process, representing the original, clean data without any added noise. Here we can take the greyscale image (translated from protein sequences) as our input. Then we select a time step *t* uniformly at random form the set {1, ……,T}. T is the total number of diffusion steps in the process. Each *t* corresponds to a specific level of noise in the diffusion process, with T being the most noise. Furthermore, to finish the diffusion process, we take a noise vector ϵ, which is a noise vector sampled from a standard multivariate normal distribution with mean 0 and covariance matrix *I* (the identity matrix). This noise vector is used to perturb the data as part of the diffusion process. Lastly, there is the core of the training algorithm. It defines the objective for the gradient descent optimization. The model, parameterized by θ, tries to predict the noise ϵ that was added to the original data x_0 at time *t*. The function ϵ_θ takes an input the noisy version of x_0 at time t, which is a mix of the scaled original data $\sqrt{\alpha_t} x_0$ and scaled noise $\sqrt{1 - \alpha_t}\epsilon$, and the time step *t*. The loss function is the mean square error between the actual noise ϵ and the predicted noise ϵ_θ. The above is the algorithm for the forward denoising process.

Table S2. Reverse diffusion process.

**Algorithm 2 Sampling**
Repeat
$x_T \sim \mathcal{N}(0, I)$
for $t = T, \ldots, 1$ do
z = $\begin{cases} \sim \mathcal{N}(0, I) & \text{if } t > 1, \\ 0 & \text{otherwise.} \end{cases}$
$x_{t-1} = \frac{1}{\sqrt{\alpha_t}}\left(x_t - \frac{1-\alpha_t}{\sqrt{1-\bar{\alpha}_t}}\epsilon_\theta(x_t, t)\right) + \sigma_t z$
end for
return x_0

Same as the previous algorithm: initialize the sampling process, where x_T is randomly sampled from a multivariate normal distribution with a mean of 0 and covariance is the identity matrix *I*. This means that at time step T (the final step of the diffusion sequence), we start the reverse reconstruction process from a pure noise state. Then there is a loop statement that starts from time T in reverse order until 1, gradually removing noise to approximate the original data x_0. T is the total number of steps in the diffusion process. Next is the conditional judgment: At each time step *t*, if *t*>1 then z is the noise vector sampled from the standard normal distribution $\mathcal{N}(0,1)$. In the final step (*t*=1), z is set to 0, indicating that we will not introduce any new noise in the final step. The most important step: Given the current noise data x_t and time step *t*, use the trained model ϵ_θ to predict the noise added in the current step. Then subtract the estimated noise from x_t to approximate the data of the previous time step x_{t-1}. Here, α_t and σ_t are coefficients that control the noise level, varying with *t*. The final return is the gradually denoised data x_0, which is progressively reconstructed from the initial pure noise state x_t and should be close to the original data.

Table S3. Parameters of the ESM2 model

ESM2 Model	Layers	Params	Dataset	Embedding Dim
esm2_t6_8m_UR50D	6	8M	UR50/D2021_04	320
esm2_t12_35M_UR50D	12	35M	UR50/D 2021_04	480
esm2_t30_150m_UR50D	30	150M	UR50/D 2021_04	640
esm2_t33_650m_UR50D	33	650M	UR50/D 2021_04	1280
esm2_t36_3b_UR50D	36	3B	UR50/D 2021_04	2560
esm2_t48_15b_UR50D	48	15B	UR50/D 2021_04	5120

The ESM2 model utilized in this paper is ESM2_t12_35M_UR50D, featuring 12 layers, a parameter size of 35 M, and an embedding dimension of 480.

Figure S1. Kinetic Association-Dissociation Curve.

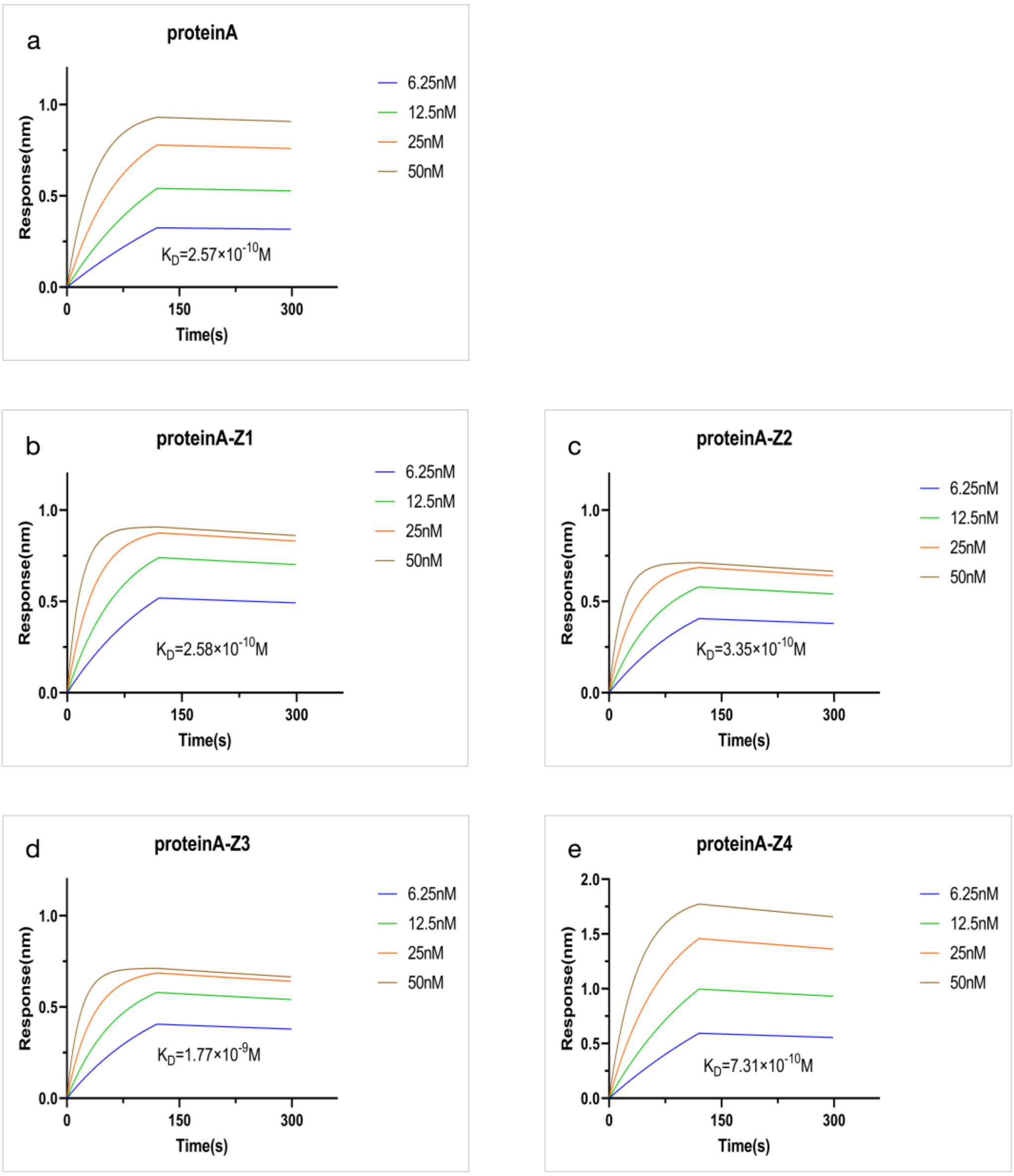

(a),(b),(c),(d),(e): Protein A as well as mutants of protein A were immobilized on His sensor (Catalog#18-0038) were purchased from SARTORIUS, and their binding and dissociation from human IgG4 mAb1 were measured at different concentrations (6.25 nM, 12.5 nM, 25 nM and 50 nM). The results clearly showed that the affinities were Z1, Z2, Z4 and Z3 in descending order. It is noteworthy that the affinity of the modified Z1 mutant was comparable to that of protein A
